# Effects of Functional Electrical Stimulation Cycling of Different Duration on Viscoelastic and Electromyographic Properties of the Knee in Patients with Spinal Cord Injury

**DOI:** 10.3390/brainsci11010007

**Published:** 2020-12-23

**Authors:** Antonino Casabona, Maria Stella Valle, Claudio Dominante, Luca Laudani, Maria Pia Onesta, Matteo Cioni

**Affiliations:** 1 Laboratory of Neuro-Biomechanics, Department of Biomedical and Biotechnological Sciences, School of Medicine, University of Catania, 95123 Catania, Italy; casabona@unict.it (A.C.); dominante84@hotmail.it (C.D.); llaudani@cardiffmet.ac.uk (L.L.); mcioni@unict.it (M.C.); 2 Residency Program of Physical Medicine and Rehabilitation, Department of Biomedical and Biotechnological Sciences, University of Catania, 95123 Catania, Italy; 3 Cardiff School of Sport and Health Sciences, Cardiff Metropolitan University, Cardiff CF5 2YB, UK; 4 Spinal Cord Unit, Cannizzaro Hospital, 95126 Catania, Italy; mp.onesta@gmail.com; 5 U.O.P.I. Gait and Posture Analysis Laboratory—A.O.U. Policlinico Vittorio Emanuele, 95123 Catania, Italy

**Keywords:** kinematics, spinal cord injury, paraplegia, spatiotemporal analysis, electromyography, regression analysis, stiffness, viscosity, pendulum test, neurologic disorders

## Abstract

The benefits of functional electrical stimulation during cycling (FES-cycling) have been ascertained following spinal cord injury. The instrumented pendulum test was applied to chronic paraplegic patients to investigate the effects of FES-cycling of different duration (20-min vs. 40-min) on biomechanical and electromyographic characterization of knee mobility. Seven adults with post-traumatic paraplegia attended two FES-cycling sessions, a 20-min and a 40-min one, in a random order. Knee angular excursion, stiffness and viscosity were measured using the pendulum test before and after each session. Surface electromyographic activity was recorded from the rectus femoris (RF) and biceps femoris (BF) muscles. FES-cycling led to reduced excursion (*p* < 0.001) and increased stiffness (*p* = 0.005) of the knee, which was more evident after the 20-min than 40-min session. Noteworthy, biomechanical changes were associated with an increase of muscle activity and changes in latency of muscle activity only for 20-min, with anticipated response times for RF (*p* < 0.001) and delayed responses for BF (*p* = 0.033). These results indicate that significant functional changes in knee mobility can be achieved by FES-cycling for 20 min, as evaluated by the pendulum test in patients with chronic paraplegia. The observed muscle behaviour suggests modulatory effects of exercise on spinal network aimed to partially restore automatic neuronal processes.

## 1. Introduction

The spinal cord is a central vector of neural signals that connects the body to the brain. Complete spinal cord transection leads to interruption of these signals, with irreversible and permanent impairment of sensory and motor functions. When the lesion is located below the first thoracic vertebra it causes paraplegia, i.e., a paralysis of the lower limbs. In paraplegic persons, the evolution of spinal damage is characterized by an early spinal shock characterized by flaccidity and either reduced or completely suppressed spinal reflexes, followed by a hypertonic phase with spasticity and exaggerated reflexes produced by the lack of suprasegmental motor control [1].

In the chronic phase following complete spinal cord injury (SCI), patients with paraplegia develop serious degenerative consequences on the musculoskeletal system, involving bones, muscles, and ligaments [2,3]. In particular, patients can experience a variety of muscle problems from spasticity, clonus and flexor spasms to soft and limp muscles lacking tone. These conditions result in a loss or limited mobility of lower limbs that gives rise to a sedentary lifestyle in a wheelchair, with repercussions on cardiovascular and metabolic functioning [4,5].

A growing number of studies have shown that regular physical exercise has a positive impact on the quality of life of people with SCI [6,7,8]. Over the last decades, physical exercise has been associated with muscle functional electrical stimulation (FES) resulting as an effective rehabilitation approach [9,10,11]. The FES technique is based on the application of electrical impulses to the muscles while the relevant body segments are in motion, with a double purpose: (i) preventing damage related to inactivity (atrophy, retraction, joint stiffness, or instability) of the muscles, and (ii) producing functional movements, otherwise impossible, through the muscular contraction elicited by electrical stimulation. Structural and metabolic improvements have been demonstrated by FES training with positive effects on contractile properties [12,13], muscle microcirculation [14], and oxidative capacities [13]. Moreover, atrophies and alterations related to disuse are prevented and the induced changes are reversible [15].

Exhaustive physiological benefits have been ascertained for persons with SCI when FES is connected with a motorized cycle ergometer (FES-cycling). This hybrid protocol enhances levels of fitness, increasing muscle mass [2], and provides appropriate cardio-metabolic [16] and pulmonary [17] prevention programs. The integration of natural movements with direct muscle stimulation, as in FES-cycling protocol, may have important implication in producing spinal cord and muscle plasticity [18]. It is recognised that motor activity intensifies production of specific neurotrophic factors in muscle and spinal cord, both in conditions with intact spinal cord [19,20] and following spinal cord transection [21,22]. Several studies have shown that FES can help to convert type II muscle fibers back to type I fibers that are normally reduced in paraplegic patients following inactivity [23,24]. In addition to muscle remodelling, a consistent spinal circuitry plasticity may be triggered from muscular and joint proprioceptors activation, following natural movements and muscle stimulation [18,25,26,27]. This can be particularly important for patients with complete spinal lesion, since peripheral afferents represent the only possible natural source for neuronal stimulation.

In this study we evaluated the effect of FES-exercise on knee mobility in chronic SCI patients with complete impairment of motor function, quantifying biomechanical and muscle activity changes by means of the pendulum test. Typically, pendulum test has been applied on subjects with SCI to assess the level of spasticity from biomechanical data, such as angle excursion or level of stiffness [10,11,28,29,30,31]. However, this test provides useful information on knee mobility also in presence of low levels of muscle tone [32,33,34] or connective disorders [35]. In these cases, patterns of muscle activity can be detected, providing insights on neuronal adaptations [32,33]. To our knowledge, only Popovic-Maneski et al. [11] showed data on muscle activation during a pendulum test in chronic SCI patients, but they did not elaborate the muscle activity, analysing only the kinematics data to support the sensibility of the pendulum test in capture low level of spasticity.

Thus, one aim of this study was to verify the sensibility of the pendulum test in obtaining more comprehensive characterization of the effect of FES-cycling exercise on knee mobility in SCI patients with chronic flaccid paralysis, including both knee joint biomechanics and muscle response analysis.

Another aim of this study was to identify the “dosage”, in terms of exercise duration, sufficient to achieve beneficial effects by comparing a 20-min and a 40-min exercise session. To our knowledge, no study has investigated the minimal duration of exercise required to obtain benefit from FES in paraplegic patients. This is an important practical issue since FES-training can induce fast muscle fatigue, due to the non-physiological recruitment of motor units in which type II fibres are recruited first [36,37] and/or to synchronous stimulation of mixed fibres [38].

We hypothesize that FES-cycling exercise may reduce the muscle flaccidity in SCI patients, producing an increase in knee stability associated with an augmented level of stiffness. It remains to be ascertained to what extents this change depends on the increase in muscle activity and whether it will be present already after 20 min of exercise.

## 2. Material and Methods

### 2.1. Participants

This study was carried out on seven young adults (6 men and 1 woman) with post-traumatic paraplegia occurred a minimum of 3 years before testing. Participants were evaluated with the ASIA impairment scale (AIS) [39]. All of them were affected by paraplegia with complete lack of motor function below the level of lesion (see Table 1). Anthropometric characteristics of participants are outlined in Table 1. Muscle spasticity was graded through the modified Ashworth scale (MAS) [40]. Each participant showed a value of MAS = 0, corresponding to no increase of muscle tone. Exclusion criteria regarded any cardio-respiratory disorder or musculoskeletal disorders that would preclude the ability to cycle. As a result, three out ten patients initially enrolled were excluded from the study due to the following factors: heart disease, pressure injuries or metal implants in the lower limbs, severe osteoporosis.

All volunteers provided written informed consent before they participated in the study. The research project was approved by the local ethics committee of Catania University Hospital “Policlinico-Vittorio Emanuele” (n° 115/2019/PO, date of approval 3 December 2019), and all procedures were performed according to the Declaration of Helsinki of 1975, revised in 2013.

### 2.2. Apparatus and Procedures

#### 2.2.1. FES-Cycling Exercise

Patients were novices with FES-cycling and conducted a sedentary life. Each participant was required to attend two FES-cycling sessions: one session lasting 20 min, and another session lasting 40 min. The two sessions were separated by one week of rest and the order of sessions was randomized between participants. The randomization of the sessions permitted to reduce the variability due to uncontrolled factors, such as the effect of exercise on the arthro-muscular integrity that is difficult to monitor in these patients.

A motorized leg pedal exerciser (RECK MOTOmed viva 2, RECK-Technik GmbH & Co. KG, Betzenweiler, Germany) was used to allow safe and reliable cycling movements of the lower limbs, while applying electrical stimuli to the lower limb muscles using a specialist FES technology (CHINESPORT, RehaStim—RehaMove, Udine, Italy).

During the exercise session, patients were sitting in front of the device on their wheelchair with flexible stimulating electrodes placed over their rectus femoris (RF), biceps femoris (BF), and gluteus maximus of both lower limbs. Once the cycling exercise started, the pedal exerciser + FES-technology system perceived the position of the foot pedal and stimulated the muscles in the correct sequence to produce a fluid cycling movement. Each channel delivered up to 130 mA of electrical stimulation, with charge-balanced biphasic electrical current and amplitude between 50 and 400 μs. Each exercise session included: (i) an initial warm-up phase lasting 2 min, at a speed of 20 rpm (where rpm is the angular speed of the engine in revolutions per minute); (ii) an active phase, with electrical stimulation for 20 or 40 min, with a target speed set at 40 rpm; (iii) a cool down phase of 1 min at a speed of 20 rpm.

As the patients were unable to power the bike actively, the motorized system of the bike assisted them with motor power during all steps protocol stages, including the transition from rest to exercise and a slower (20 rpm) to a faster (40 rpm) cadence.

Tracking heart rate was performed at the beginning and at the end of the exercise session.

#### 2.2.2. Functional Assessment of Knee Joint Biomechanics

The instrumented pendulum test was used to evaluate knee joint biomechanics and electromyographic activity of the lower limb muscles, before and after each exercise session in all participants. The typical duration of time from the end of each FES-cycling exercise and the starting of the pendulum test measurements was about 20 min. The test allows a quantitative analysis of the kinematics of knee joint during passive swings of leg about the gravitational resting position; in addition, by a combination of kinematic and anthropometric data, the pendulum test provides estimates of joint viscoelastic properties.

**Figure 1:**
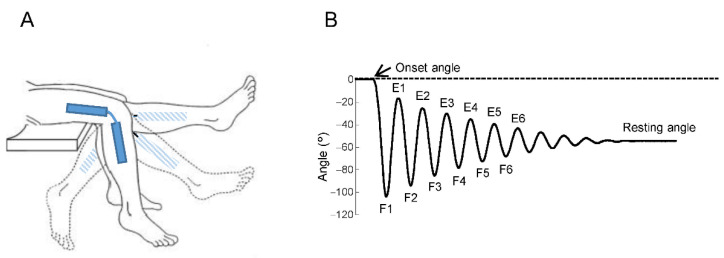
Experimental setup and typical kinematic trajectory. (**A**) Passive limb oscillations during pendulum test. The extended leg represents the starting position and the flexed leg is the rest final position. An electrogoniometer with two arms located one to the thigh and the other to the leg, recorded the angular displacements of the limb. Two couples of surface electrodes recorded the EMG activity of Rectus Femoris and Biceps Femoris, respectively. (**B**) Knee angular displacement in a healthy subject during flexion-extension movements showing onset angle, resting angle and the first 6 cycles with flexions (F1, F2,…, F6) and extensions (E1, E2,…, E6).

After receiving procedure demonstrations, the patients were placed in a safe sitting position with a back support and with the trunk inclined about 45° from the horizontal plane. The same examiner for all patients raised the leg to the extended position and then released it in order that the limb was passively oscillating, alternating flexions and extensions until stopping in the rest position (Figure 1A). This test procedure was repeated ten times, with a short pause inter-trial of about 1–2 min for a total duration of about 20 min.

Angular displacements of the knee joint during the oscillatory motion of the right leg were obtained using a biaxial electrogoniometer (Biometrics Ltd., Gwent, UK), with the proximal axis attached to the outer part of thigh and the distal axis adherent to the outer part of shank and its location over the skin was drawn to re-apply the electrogoniometer in the same position after the exercise.

After an appropriate skin preparation to reduce the electrode-skin impedance, a pair of bipolar Ag-AgCl electrodes (ARBO H 124, Kendall™ Electrodes) was attached over the RF and BF muscles, according to SENIAM recommendations [41]. These surface electrodes were left in place during the exercise testing. Both joint angle and EMG data were collected and synchronized using a portable electromyography system (PocketEMG by Bioengineering Technology and System, BTS, Garbagnate Milanese, Italy), with a sampling frequency of 1000 Hz.

### 2.3. Measurements and Estimates

#### 2.3.1. Kinematic Measurements

The kinematic measurements were elaborated to obtain the following parameters (Figure 1B): onset angle that corresponds to the angle with respect to the horizontal, before the beginning of the first oscillation; resting angle which corresponds to the angle in the rest position at the end of the oscillations; angles of reversal at the end of the first 6 flexion (hemicycles *F1–F6*) and extension (hemicycles *E1–E6*) oscillations.

Subsequently, kinematic and anthropometric data were combined to estimate the viscosity (*B*) and stiffness (*K*) coefficients for the first six flexions (*F1–F6)* and extensions (*E1–E6)* of the knee joint.

#### 2.3.2. Viscoelastic Estimation

The estimates of *B* and *K* have been quantified considering that these two factors contribute to the motion of a physical pendulum according to the following equation:

(1) $$J\ddot{\theta }+B\dot{\theta }+K\theta +mgl_{c}\sin\theta =0$$

where *θ* represents the angle of oscillation of the leg with respect to the knee; *J* is the moment of inertia of the leg-foot complex with respect to the axis of rotation around the knee; *B* is the viscosity coefficient; *K* the stiffness coefficient; *mgl_c_* factor represents the moment of rotation of the knee produced by gravity (gravitational torque), where *m* is the mass of the leg-foot complex; *lc* is the distance between the centre of mass (*COM*) of the leg-foot complex and the axis of rotation around the knee; *g* is gravity acceleration. In the above equation, the terms *B* and *K* are the only unknown, while all the others are acquired from the data collected during the experimental activity or from specific anthropometric tables [42].

From solving of Equation (1) it is possible to extract the values of *B* and *K* for each single point of the oscillatory trajectory of the leg. In this study, *B* and *K* were calculated for the first 12 hemicycles, i.e., six peaks in flexion and six in extension. Viscoelastic data were normalized with respect to the gravitational torque. To this end, the values of K and B, computed as in Casabona et al. [32], were divided by the gravitational torque. By this procedure, K and B were normalized with respect to the torque produced by the acceleration of gravity (*g*) acting on the individual anthropometric measures, i.e., leg-foot mass (*m*) and the distance of the centre of mass of the leg-foot segment from the knee axis (*l_c_*).

A previous study from our laboratory shows all calculation steps [32].

#### 2.3.3. Electromyographic Processing

The EMG signal, once amplified and sampled, was filtered offline with a high pass filter (20 Hz cut-off, 2 pass, 2nd order Butterworth), to improve the quality of data removing possible movements artefacts. After full-wave rectification of the raw EMG signal, the EMG areas during the first six flexions and extensions for each trial of the pendulum test, were obtained by computing the integral of the EMG signals filtered by a low-pass filter (10 Hz cut-off, 2 pass, 2nd Butterworth, [42]).

The latencies of EMG responses were measured with respect to the onset of the first flexion. To identify the onset of the EMG burst we used the nonlinear Teager–Kaiser energy operator (TKEO). This method considers both amplitude and frequency and computes the energy of the EMG signal and several authors have demonstrated that this method improves the signal-to-noise ratio and increases the accuracy of the EMG onset detection [43]. We established the onset of muscle activation from temporal trend of changes in each trial. In particular, for each EMG signal, the TKEO was applied on the 20 Hz high pass filtered raw signal and the TKEO output was full wave rectified. To find the threshold that allowed to determine the onset time over the TKEO domain, we used formulas and procedures present in our previous work [44].

Kinematic data were normalized with respect to the measure recorded before the first exercise session, while the EMG signals were normalized with respect to the maximum value of EMG area over the ten repetition of each pendulum test.

All signals analyses and the computations were performed by using Matlab version R2019a (Mathworks Inc., Natick, MA, USA).

#### 2.3.4. Statistical Analysis

Measurements related to kinematics and viscoelastic characteristics of the knee were preliminarily submitted to the Shapiro-Wilk test to verify the presence of a normal sample distribution. In addition, Levene’s test for equality of sample variances were performed to validate the use of parametric statistics on a small sample.

Statistical comparisons between the different experimental conditions were made using the three-way analysis of variance (ANOVA) for repeated measurements, considering the effect of exercise (pre vs. post exercise measurements), the hemicycles (F1–F6 and E1–E6), duration of exercise (20 vs. 40-min) and the effect of the interaction among the factors. The magnitude of the effect, i.e., the portion of total variability attributable to the effect of each factor, was evaluated by partial η^2^ (η_p_^2^). Post hoc pairwise analysis was performed applying t-test with Bonferroni correction.

In order to establish the relationships between viscoelastic measurements and EMG activity and between EMG and muscle latencies, linear correlations analyses were performed computing Pearson’s correlation coefficient (*r*).

For all the statistical tests, the significance level α was established at 0.05. Statistical analyses were implemented by using SPSS version 20.0 (SPSS, Inc., Chicago, IL, USA, IBM, Somers, NY, USA).

## 3. Results

**Figure 2:**
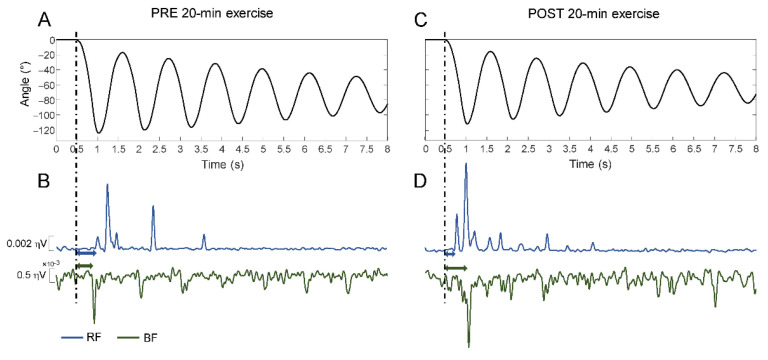
Kinematics and electromyography illustrated by means of a representative example. Kinematic trajectory and rectified EMG activity of Rectus Femoris muscle (blue) and Biceps Femoris muscle (green) recorded in a patient before 20-min exercise duration (**A**,**B**) and after 20min exercise duration (**C**,**D**).

Figure 2 shows the knee joint angular displacements during the first six oscillations and simultaneous muscle activation, for a representative subject.

The example retraces the most typical behaviors observed across the persons with SCI. The panels show one trial before to start (panels A and B) and after (panels C and D) 20-min exercise. In the post exercise period, the angular excursions decreased while the EMG activity increased in both RF and BF muscles. Notably, following the 20-min exercise, most of the changes in amplitude occurred during the first flexion, with latency of EMG responses shorter for the RF muscle and longer for the BF compared to baseline measures before the exercise.

This scheme resembles the statistical results described in the following sections.

### 3.1. Biomechanical Parameters

**Figure 3:**
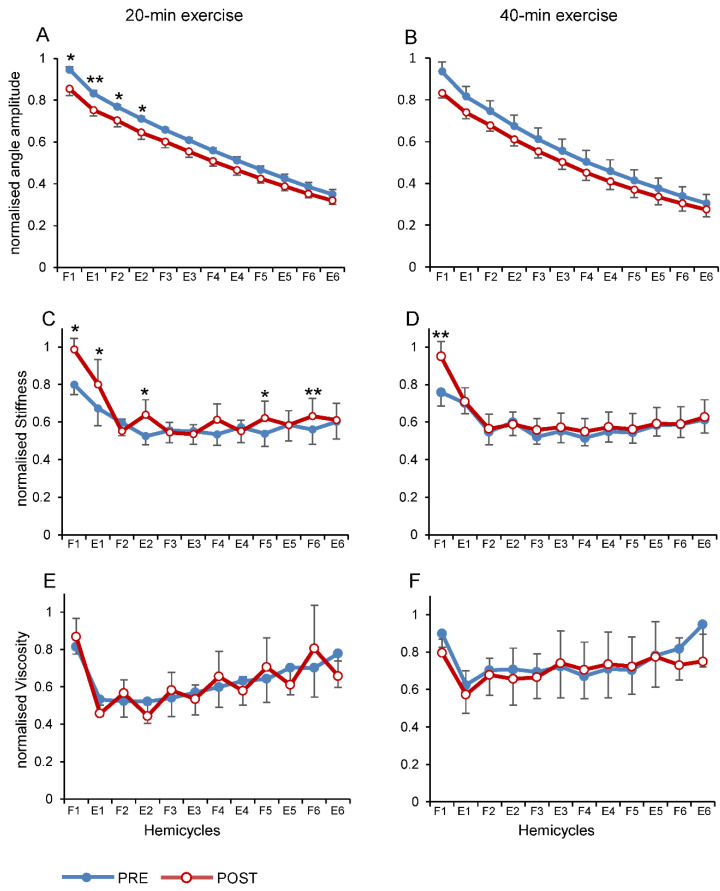
Effects of exercise on the variations of kinematic and dynamic parameters over the hemicycles and before and after the sessions of practice. Changes in amplitude of knee joint angle (**A**,**B**), stiffness (**C**,**D**) and viscosity (**E**,**F**) when measurements were obtained before (blue line) and after (red line) 20-min exercise (left panels) and before and after 40-min exercise (right panels). Data are expressed as grand average and standard error collected from all the participants. Abbreviations and symbols: F, flexion; E, extension; * *p* < 0.05; ** *p* < 0.01 are referred to pre vs. post comparisons.

Figure 3 illustrates the changes in biomechanical parameters for each hemicycle during the first six cycles. Amplitude of knee joint angle (Figure 3A,B) progressively decreased over hemicycles (F_1.23,7.37_ = 223.02, *p* < 0.001, η^2^_p_ = 0.97) and it was reduced following both 20- and 40-min exercises compared to the respective baseline measures. In particular, there was a tendency towards significance for the exercise duration (F_1,6_ = 5.89, *p* = 0.051, η^2^_p_ = 0.50), and a significant interaction between exercise duration and hemicycles (F_1.54,9.24_ = 6.15, *p* = 0.025, η^2^_p_ = 0.51). Noteworthy, this interaction depended mainly on the larger differences between pre- and post-exercise during the initial hemicycles rather than during the following hemicycles. In fact, local pairwise comparisons performed within each exercise duration showed significant differences between pre- and post- exercise for the first four hemicycles during 20-min exercise (Figure 3A) and a tendency toward significance for the first hemicycle during the 40-min exercise (Figure 3B; *p* = 0.055).

No significant effects were detected for the duration of exercise and for the other factor interactions.

Stiffness of the knee joint increased after exercise (Figure 3C,D), showing a statistical pattern similar to those observed for the changes in angle amplitude. In particular, there was a main effect of the hemicycles (F_1.54,9.22_ = 11.39, *p* = 0.005, η^2^_p_ = 0.66) and a significant interaction between exercise duration and hemicycles (F_2.53,15.21_ = 7.47, *p* = 0.004, η^2^_p_ = 0.55), with no significant differences for the other factors. The most important changes of stiffness over time occurred during the first two hemicycles, indicating that a main contribution for the effect of hemicycles come from the first flexion and extension. In fact, joint stiffness showed a significant increase specifically in the first two hemicycles and in some of the following hemicycles for the 20-min exercise duration (Figure 3C), and only in the first hemicycle for the 40-min exercise duration (Figure 3D).

No significant changes across all the factors were observed for the viscosity (Figure 3E,F).

Since most changes in both angle amplitude and stiffness over hemicycles and pre vs. post exercise occurred mainly during the first two hemicycles, we applied a three-way ANOVA reducing the number of hemicycles to the first two. The results showed a main effect of exercise for both angle amplitude (F_1,6_ = 11.54, *p* = 0.015, η^2^_p_ = 0.66) and stiffness (F_1,6_ = 18.42, *p* = 0.005, η^2^_p_ = 0.75). Moreover, there was an interaction between duration of exercise and hemicycles for the stiffness (F_1,6_ = 8.64, *p* = 0.026, η^2^_p_ = 0.59) indicative of a different behaviour between 20- and 40-min durations in the first two hemicycles. As shown in Figure 3C,D, the 40-min exercise protocol showed a significant increase during the flexion hemicycle only, whereas the 20-min protocol showed an increase during both flexion and extension hemicycles.

### 3.2. Electromyographic Parameters

**Figure 4:**
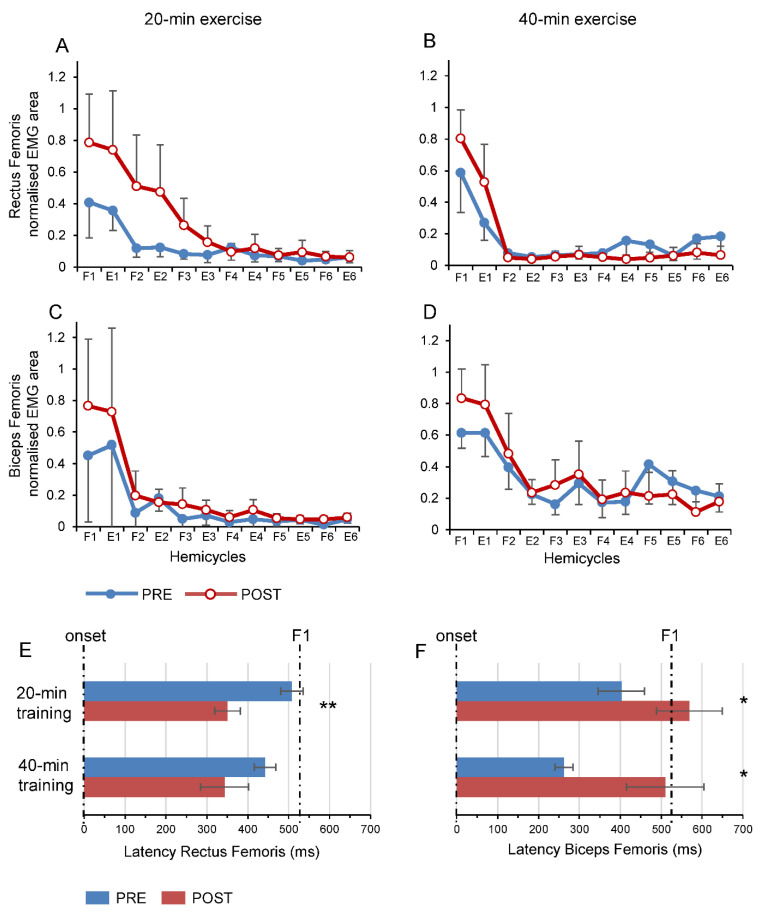
Effects of exercise on EMG activity over the hemicycles and before and after the sessions of practice. Changes in EMG amplitude for Rectus Femoris (**A**,**B**) and Biceps Femoris (**C**,**D**) when recording, were performed before (blue line) and after (red line) 20-min exercise (left panels) and before and after 40-min exercise (right panels). Comparison of the latencies of EMG activity of Rectus Femoris (**E**) and Biceps Femoris (**F**), before and after exercise. Vertical dotted lines represent the onset and the peak of the first flexion, respectively (F1). Data are expressed as grand average and standard error collected from all the participants. Abbreviations as in Figure 3. * *p* < 0.05; ** *p* < 0.01 are referred to pre vs. post comparisons.

Figure 4A,D illustrates the trends in EMG activity following both exercise duration protocols over each hemicycle. The main increase in EMG activity following the exercise mainly occurred over the first flexion and extension hemicycles (Figure 4A,D). Indeed, there was a main effect of the hemicycles for the RF muscle (F_1.40,8.42_ = 6.05, *p* = 0.03, η^2^_p_ = 0.50), but no differences were detected for both RF and BF muscles with respect to the two exercise protocols. Visual inspection of Figure 4A,D suggests that the lack of significant differences was due to a reported high variability in EMG activity between participants.

The temporal characteristic of EMG bursts showed relevant significant changes (Figure 4E,F). The onset of RF EMG activity (Figure 4E) was significantly reduced after 20-min exercise, with an average reduction of 157 ms (−31%; *p* < 0.001), but not after 40-min exercise (average reduction of 99 ms; −22%; *p* = 0.107). Conversely, the EMG activity of BF (Figure 4F) showed a significant increase in the onset time after both 20-min exercise (average increase of 166 ms; +41%; *p* = 0.033) and 40-min exercise (average increase of 248 ms; +95%; *p* = 0.03). Visual inspection of Figure 4E,F shows that onset time of the RF muscle shifted sharply towards the beginning of the first flexion, while the onset time of BF varied within a region on the boundary between the end of the first flexion and the begin of the first extension.

### 3.3. Regression Analysis

**Figure 5:**
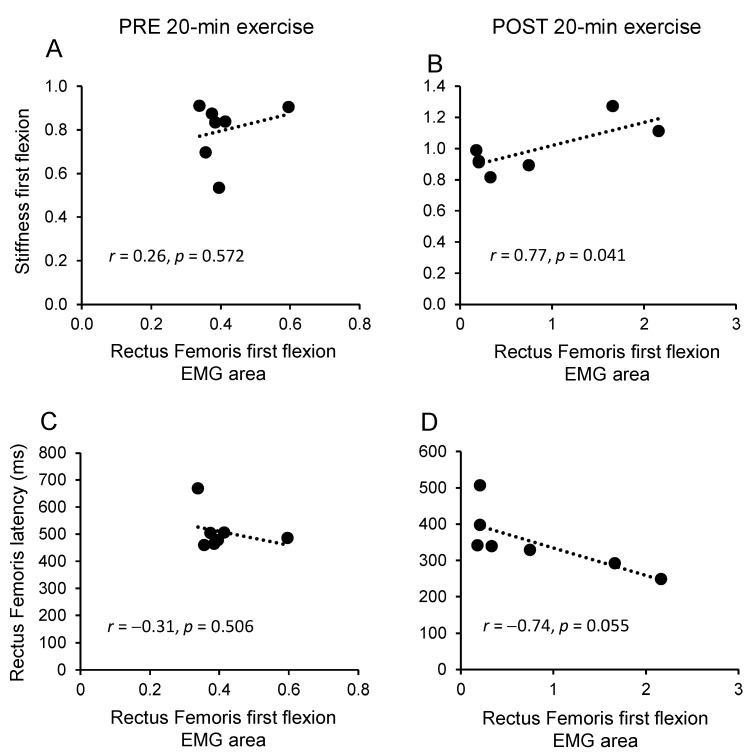
Linear regression analysis. Relationship between the changes of stiffness and EMG area of Rectus Femoris during the first flexion, before (**A**) and after (**B**) 20-min exercise. (**C**) Relationship between the changes in latency of Rectus Femoris and EMG area of the same muscle, during the first flexion before (**C**) and after (**D**) 20-min exercise. Abbreviations: *r*, correlation coefficient; *p*, level of significance of the correlation.

A regression analysis was conducted to quantify the contribution of the muscle activity on the increase of stiffness during the first leg flexion and extension, before and after the exercise. The only significant correlation concerned the relationships between joint stiffness in the first flexion and RF muscle activity after the 20-min exercise (r = 0.77; *p* = 0.041; Figure 5A,B). No significant correlation was found for any comparison regarding BF muscle, extension movement, or the exercise protocol of 40 min.

In addition, we verified a possible association between the changes in EMG area and in latency for both RF and BF muscles (Figure 5C,D). As for the relationship between muscle EMG and stiffness, the only notable result occurred after 20-min exercise and concerned a marginally significant correlation between the RF latency and the EMG activity of the RF during the first flexion.

## 4. Discussion

In this study, the pendulum test was used to evaluate the acute effect of FES-cycling of different durations on knee mobility in individuals with complete impairment of motor function. Following each FES-cycling session of either 20- or 40-min duration, amplitude of the knee joint angle was reduced over the pendular leg oscillations, with a parallel increase in joint stiffness during the first flexion and extension hemicycles. The increase in stiffness level during the first flexion was directly correlated with an increase in phasic EMG activity of the RF muscle only after the 20-min exercise, but not following 40-min exercise. Moreover, exercise modulated the timing of RF and BF activity burst, moving their onset close to the beginning of first flexion for the RF and close to the reversal point between flexion and extension for the BF. Interestingly, following the 20-min exercise only, these latency changes were correlated with changes in EMG amplitude of the RF.

### 4.1. The Pendulum Test as a Tool to Evaluate the Effects of FES-Cycling on Knee Mobility

The pendulum test is commonly used to estimate the level of spasticity in a number of neurological diseases [31,45,46]. However, this tool has been shown to be effective in the evaluation of knee joint mobility in conditions characterised by increased mobility, such as in Down syndrome [32,33,34] and in rheumatoid arthritis associated with neuronal-independent mobility reductions [35].

An increase of stiffness, with a drastic reduction in the amplitudes of knee excursion, has been previously reported for patients with high levels of spasticity, by using the pendulum test [10,11,28]. Considering that the patients enrolled in this study were characterized by muscle flaccidity, the reduced knee angular excursion associated with the FES-cycling exercise should not depend on spastic muscle contraction, but on changes in the complex interaction between the passive and active tissues forming the knee joint structure. Here, the observed consistent muscle activity during leg oscillations, could indicate that most of the changes in knee mobility depended on the modulation of the active components of the joint, i.e., muscle tissue.

Despite to the high variability in EMG activity which determined a lack of statistical significance, the level of muscle activation tended to increase following FES-cycling exercise, with a clear and direct correlation between RF muscle activity and joint stiffness. In fact, the increase in RF EMG area during the first flexion was strongly correlated with increase in level of stiffness following the 20-min exercise session, but not following the 40-min session. Therefore, following the 20-min FES-cycling session, the sequence of functional events that led to a reduction of the joint angle amplitude started from the modulation of the RF activity, which increased the stiffness at knee and the latter, in turn, reduced the joint excursion.

These results demonstrate the value of the pendulum test in quantifying kinematic changes and/or spasticity in people with SCI, as reported in several studies [10,11,28,29,30,31], but it can also detect both temporal and amplitude modulations of muscle activity. In this way, this test can provide data to infer about the modulation of the spinal circuits associated with training protocols.

### 4.2. Possible Spinal Neuronal Adaptations to FES-Cycling

We believe that the muscle latency and amplitude adjustments observed in the current study may depend on a functional adaptation occurred in the spinal circuits during the training. An important prerequisite to implement this adaptation after SCI is to provide spinal cord with an appropriate quantity and quality of somato-sensory afferents [18,25,26,27,47,48]. Studies to date indicate that hybrid physical interventions, with natural movement assisted by mechanical or electrical muscle stimulation, are suitable paradigms to obtain consistent muscle and joint sensory receptors activations [18,25,49].

A connection between changes in latency of muscle activation and spinal circuit modifications after SCI, was found during mechanical assisted locomotion in humans by Dietz et al. [50] and in rats by Beauparlant et al. [26]. These authors reported a long-latency activation of the Tibialis Anterior muscle in chronic SCI patients that was analogous with the timing of responses observed in the current study for the RF. This late component appeared from 6 months after the injury, with the patients showing an EMG exhaustion during locomotor activity. On the contrary, an early spinal reflex component was present around 2 months after SCI without significant muscle exhaustion during locomotion.

Dietz et al. [50] concluded that the association of a late spinal response latency with muscle exhaustion in chronic SCI patients indicates that long period of immobilization led to degradation of spinal reflex and muscle functionality. This interpretation was confirmed by Beauparlant et al. [26] that compared results from rat and humans and found that late reflex responses appeared after a long period of inactivity from the injury, in concomitance to an aberrant flux of sensory afferents, producing an inappropriate spinal network plasticity.

As our participants had not undergone any constant physical therapy protocol before and at the time of testing, the observed long latency responses could be associated to anomalous sensorimotor integration formed during long period of immobilization. In this view, the shorter responses of RF, statistically significant only for the 20-min session, may indicate that the FES-cycling exercise has orientated the sensory afferents to guide a functional restoration of spinal circuits.

To our knowledge, no previous study has reported any direct evidence that the reduction in timing of the late EMG response in chronic SCI is a mark for spinal plasticity, resulting in restoring functional motor behaviour. However, integrating the data reported by Dietz et al. [50] and Beauparlant et al. [26] with our results, the following three points could support the association between changes in muscle latency and functional adaptation of spinal circuits.

First, the late spinal responses in Dietz et al. [50] and Beauparlant et al. [26] were functionally connected with a reduction in muscle activity, while the short latency responses were present at the early stages of SCI, showing EMG amplitude similar to healthy people. The shortening in onset time of RF EMG observed in the current study was correlated with increases in the amount of EMG activity, indicating that the changes in latency were orientated towards the functional muscle restoration, as in Dietz’s schema.

Second, Dietz et al. [50] and Beauparlant et al. [26] recorded the Tibialis Anterior activity during an assisted locomotion and suggested that the late reflex response results from deterioration of the signals from the central pattern generator. In the current study, the patients performed a cycling task that requires a pattern of signals from spinal cord, with an alternation between flexions and extensions of the leg that is similar to the locomotor pattern. Considering that after the exercise, the pendulum test revealed an opposite temporal shift of the two antagonist muscles, with RF onset moving towards the beginning of the leg flexion and the BF onset toward the initiation of extension, it is reasonable to assume that this behaviour could emerge from a reorganization of the signals coming from the central pattern generator.

Finally, on the basis of the well-documented association between spinal neuroplasticy and the amount of sensory afferents to the spinal cord [26,47,48], most of the neuronal adaptations should occur during the initial hemicycles of the pendulum test, as the amplitudes of the following leg oscillations were progressively reduced, producing an ever weakening sensory signal [51]. Therefore, the presence of the muscle responses mostly during the first flexion is a further clue indicating that the RF muscle behaviour observed in this study may be associated with a remodulation of spinal circuits. Moreover, the lack in correlation between BF EMG amplitude and latency may depend on the weaker sensory signals due to the reduced amplitude of knee oscillations starting from the first extension.

We are aware that the changes in EMG activity and latency observed in this study should be temporary adaptations, since a stable remodelling of neuronal networks likely requires more than a single session of exercise. Thus, it will be important to provide a more suitable experimental design and multisession training protocols to demonstrate if the adaptations reported in this study persist for long-term, producing a plastic remodulation of the spinal network. However, the short-term adaptations observed in the present work could be a prerequisite for a successful therapy in regenerating functionality of spinal circuits.

### 4.3. Practical Implications

An important practical implication may derive from the data regarding the duration of exercise implemented in this study. In fact, although significant increasing in knee stiffness and muscle activity were present after the two periods of exercise, 20-min was more effective than 40-min exercise to originate the functional correlations among changes in knee biomechanics, EMG amplitude and latencies of muscle activity. Thus, considering the discussion reported in the previous section, the assumed spinal neuronal adaptation could be dose-dependent. Noteworthy, since these improvements were observed through the pendulum test that involves an oscillatory motion around the knee, it is reasonable to speculate that SCI patients might improve their neural/muscular activity and stiffness control during other rhythmic movements of daily life, such as assisted locomotor activities.

This should help to manage the higher fatigue rate associated with muscle activation by electrical stimulation with respect to the voluntary contraction. In fact, a reversal order of recruitment of motor unit has been reported during FES, with fast fatigable motor units activated before slow oxidative motor units [36,37]. In addition, the muscle electrical stimulation produces a massive and synchronous stimulation of all types of fibres, with a rapid glycogen depletion [37,38].

Thus, this peculiar condition imposed by FES training, enhances further the necessity to set the appropriate duration of training protocol in order to obtain an optimal result with the minimal metabolic effort.

Clinicians should be aware that dosing of FES cycling must be adapted to the anatomo-functional conditions of a chronically injured spinal cord and that a prolonged duration of exercise could be unnecessary for the effectiveness of the intervention or even harmful with respect to the risk in producing muscle exhaustion or other discomforts.

A further clinical application of our results regards stiffness and viscosity of knee of patients with chronic spinal cord injury when trained with robotic exoskeletal systems for gait rehabilitation [52]. In fact, the pre-exercise evaluation of these two parameters for each patient, according to our methodology, could give quantitative information useful to better set up the robotic programming of control of trajectories kinematics of the hip and knee and the amount of electromechanical assistance provided to the lower limbs during walking.

### 4.4. Limitations

The findings reported in this paper must be considered within the limitations of the study. A main limitation was the small sample of participants showing intrinsic inter-individual differences associated with the type and severity of the injury; this likely led to high variability and lack of statistical results for the EMG amplitude and the correlation between RF amplitude and latency. Another limitation was the lack of a control condition for the baseline data (i.e., pre-post measures with a quiet rest in between) in the present research design, which could have allowed to explore the extent of variability in the measurement of kinematic and EMG parameters. Unfortunately, due to reduced accessibility to the patients, we had to reduce the protocol timeline to the minimum necessary. Thus, we used the data collected by pendulum test at rest, before starting the first session of exercise, as reference for the kinematic normalization. Despite these limitations, the results corroborate the effectiveness of the pendulum test in quantifying functional differences in knee joint stiffness and muscular activations following a single FES exercise session in SCI patients, thus providing a solid basis for further studies on the long-term adaptations to FES training aimed at facilitating/stimulating selective neural adaptations at spinal level.

## 5. Conclusions

In this study, the pendulum test was shown to provide not only a reliable biomechanical characterization of the effects of FES-cycling on knee mobility in patients with SCI, but also to reveal muscle activation patterns that can be considered possible markers of spinal circuits adaptations to exercise. These results can be obtained through FES-cycling sessions lasting no longer than 20 min, thus ensuring an adequate control of fatigue rate in paraplegic patients. The information provided by this paper can improve the set up and monitoring of clinical interventions, but further studies are needed to establish how extensive these improvements can be and how quickly the training response can be achieved.

## Figures and Tables

**Table 1:** Anthropometric and injury-related data for participants enrolled in this study.

Patients	Age	Years Since Injury	Injury Level	AIS *	Height (cm)	Weight (kg)	Fibula Head-Ground (cm)
# 1	30	11	T7	A	180	75	45
# 2	27	3	T6	A	170	60	42
# 3	29	16	T6	B	177	58	45
# 4	36	8	T4	B	181	76	46
# 5	29	9	T12	A	170	83	42
# 6	35	12	T8	A	166	42	39
# 7	40	14	T5	A	185	85	46

* AIS: Asia Impairment Scale: A, no sensory or motor function is preserved; B, sensory but not motor function is preserved.

## Data Availability

The data that support the findings of this study are available on request from the corresponding author, M.S.V. The data are not publicly available due to containing information that could compromise the privacy of research participants.
